# Types of Inner Dialogues and Functions of Self-Talk: Comparisons and Implications

**DOI:** 10.3389/fpsyg.2020.00227

**Published:** 2020-03-06

**Authors:** Piotr K. Oleś, Thomas M. Brinthaupt, Rachel Dier, Dominika Polak

**Affiliations:** ^1^Institute of Psychology, The John Paul II Catholic University of Lublin, Lublin, Poland; ^2^Department of Psychology, Middle Tennessee State University, Murfreesboro, TN, United States

**Keywords:** inner dialogue, intrapersonal communication, self-talk, inner speech, identity, self-regulation

## Abstract

Intrapersonal communication occurs in several modes including inner dialogue and self-talk. The Dialogical Self Theory (Hermans, 1996) postulates a polyphonic self that is comprised of a multiplicity of inner voices. Internal dialogical activity implies an exchange of thoughts or ideas between at least two so-called “I-positions” representing specific points of view. Among the functions served by self-talk are self-criticism, self-reinforcement, self-management, and social assessment (Brinthaupt et al., 2009). This paper explores the relationships among different types of internal dialogues and self-talk functions. Participants included college students from Poland (*n* = 181) and the United States (*n* = 119) who completed two multidimensional measures of inner dialogue and self-talk. Results indicated moderately strong relationships between inner dialogue types and self-talk functions, suggesting that there is a significant overlap between the two modes of communication. We discuss several implications of these findings for exploring similarities and differences among varieties of intrapersonal communication.

## Introduction

Intrapersonal communication occurs in several modes and includes research on a wide range of processes and behavioral domains (see this Research Topic). Two such modes are self-talk and internal dialogue. With respect to self-talk, psychologists originally described inner and private speech in the context of developmental processes including the affinity between speaking and thinking (Vygotsky, 1962). Although inner dialogues had long been recognized by philosophers such as Thomas Aquinas and Saint Augustine, and by writers, poets, and other thinkers, formal psychological theorizing about such phenomena was only recently introduced at the end of the 20th and beginning of the 21st century (Hermans and Kempen, 1993; Markova, 2005).

The possible relationship and mixing of these two phenomena occurs within theory and empirical research. For example, according to Kross et al. (2014), “Self-talk is a ubiquitous human phenomenon. We all have an internal monologue that we engage in from time to time” (p. 321). How people engage in internal monologues (or dialogues) and self-talk is likely to vary. For example, people might instruct themselves to “Try again” or relax themselves by saying “Don’t worry.” In a different context, one might ask oneself “What can I do?” or “Are my talents and knowledge enough to argue in a coming debate?”

These examples of self-talk can also involve dialogic features. From the perspective of Dialogical Self Theory (Hermans, 1996; Hermans and Gieser, 2012), people can take at least two points of view or “I-positions” within their intrapersonal communication. We might discuss in our minds multiple options, like a fiddler on a roof: “on the one hand …, but on the other hand …” Such dialogues can show even greater complexity and detail. For example, a man might imagine how a request for a divorce will affect his spouse, how she would likely respond to that request, whether he should reconsider based on her likely response, etc. This kind of inner dialogue involves posing questions on behalf of the imagined partner and giving answers.

As the previous example suggests, an inner monologue can easily evolve into an internal dialogue between two subjects inside one’s mind—between different parts of oneself or between oneself and the imagined partner. In other words, there may be qualitative and quantitative differences in the nature of self-talk and internal dialogues. Self-talk appears to involve basic self-regulatory functions like self-control or self-direction (“Try again”), whereas internal dialogues involve more extended communicative functions (“When I say X, she will answer Y”). In the present study, we aimed to explore the degree of overlap between these two forms of intrapersonal communication.

For our purposes, self-talk can be defined as “self-directed or self-referent speech (either silent or aloud) that serves a variety of self-regulatory and other functions” (Brinthaupt, 2019, para. 7). Internal dialogical activity is defined as “engagement in dialogues with imagined figures, the simulation of social dialogical relationships in one’s own thoughts, and the mutual confrontation of the points of view representing different I-positions relevant to personal and/or social identity” (Oleś and Puchalska-Wasyl, 2012, p. 242).

Most definitions of self-talk and inner speech assume that, in this form of intrapersonal communication, both sender and recipient represent the same person (e.g., Fernyhough, 2016). In contrast, inner dialogical activity does not imply that. Inner dialogues refer to various forms of intrapersonal communication where different voices can represent not only the self but also close persons, imagined friends, lost relatives and spouses, teachers and mentors, media stars, voices of culture, and others (Hermans, 1996). Self-talk can be just a single word, comment, or command without any answer or an extended “conversation,” while mutual exchange of expressions is an essence of the internal dialogue.

Whereas everyday self-regulation is an important feature of self-talk (Brinthaupt et al., 2009), internal dialogical activity emphasizes confrontation or integration of different points of view as a way to help a person understand new or strange experiences. In other words, self-talk seems to occur in reaction to or anticipation of specific events or circumstances, whereas inner dialogue appears to involve more reflective or contemplative kinds of intrapersonal communication. Furthermore, inner dialogues frequently involve a person’s identity (e.g., Bhatia, 2002; Batory, 2010), whereas self-talk seems to apply to identity questions only indirectly.

In this paper, we first describe theoretical and research conceptions of self-talk and inner dialogical activity. We then propose possible relationships between these two forms of intrapersonal communication. Next, we report the results of a study that compares total and subscale scores of these constructs. The nature of the relationship between inner dialogues and self-talk has important implications for the phenomenon of intrapersonal communication. We discuss some of these implications in the conclusion of the paper.

### Self-Talk and Its Different Functions

Most approaches to studying self-talk assume that it encompasses self-referent or self-directed speech. Research examines several variants of the phenomenon, including positive and negative self-statements (Kendall et al., 1989), silent self-talk (i.e., inner speech) (McCarthy-Jones and Fernyhough, 2011), and out loud self-talk (i.e., private speech) (Duncan and Cheyne, 1999). Self-talk research has long been popular in the domains of clinical (e.g., Schwartz and Garamoni, 1989), sport and exercise (e.g., Hardy, 2006), developmental (e.g., Diaz and Berk, 1992), educational (e.g., Deniz, 2009), and personality (e.g., Brinthaupt et al., 2009) psychology.

Extensive research explores how and why people talk to themselves and whether variations in self-talk content result in different effects on the speaker. Among the self-talk functions are general self-regulation (e.g., Mischel et al., 1996; Carver and Scheier, 1998), self-distancing (Kross et al., 2014), providing instruction and motivation (Hatzigeorgiadis et al., 2011), and self-awareness, self-evaluation, self-knowledge, and self-reflection (White et al., 2015; Morin, 2018).

Evidence suggests that self-talk also plays a role in facilitating a variety of cognitive processes (Langland-Hassan and Vicente, 2018) including emotion regulation (Orvell et al., 2019), coping with painful experiences (Kross et al., 2014, 2017), monitoring of language development and speech production (e.g., Pickering and Garrod, 2013), and perspective taking (e.g., Fernyhough, 2009). Recent studies show that non-first-person self-talk can promote self-distancing and adaptive self-reflection (e.g., Kross et al., 2014; White et al., 2015). Referring to oneself in the third person (he/she/they) or by one’s name appears to promote coping with stressful experiences and is associated with appraising future stressors as challenges rather than threats (Kross et al., 2014, 2017). This kind of self-talk is also connected to specific forms of brain activity that constitute effortless self-control (Moser et al., 2017) and emotion regulation (Orvell et al., 2019).

A detailed functional view emerged from the development of the Self-Talk Scale (STS) (Brinthaupt et al., 2009), which measures the self-reported frequency of different kinds of self-talk. Relying on an initial pool of items assessing multiple situations where self-talk might occur and the possible common functions served by it, Brinthaupt et al. identified four broad types. The STS includes subscales on self-criticism (i.e., situations when bad things have happened to a person), self-reinforcement (i.e., relating to positive events), self-management (i.e., determining what one needs to do), and social-assessment (i.e., referring to past, present, or future social interactions).

Research on the psychometric properties of the STS supports these four factors as well as other features of the measure (e.g., Brinthaupt et al., 2009, 2015; Brinthaupt and Kang, 2014). Additional research (Morin et al., 2018) suggests that the kinds of self-talk measured by the STS are common occurrences in the everyday experience of this kind of intrapersonal communication. Thus, one way to provide an initial assessment of the relationship between the varieties of self-talk and inner dialogues is to utilize a measure that captures at least some of the possible functions served by self-talk.

### The Dialogical Self and Inner Dialogues

Bakhtin (1973) introduced the notion of the polyphonic novel with his analysis of Fyodor Dostoevsky’s literary works. That analysis showed possible splitting of the self into voices that were not exactly coherent, and each of them represented relatively autonomous points of view. According to the Dialogical Self Theory (DST) (Hermans, 1996), human consciousness functions as a similar “society of mind” containing mental representations of numerous voices of culture, family members, close friends, significant others, and other sources. These voices can engage in a variety of communications, including posing questions and answers to, and having agreements and disagreements with, each other (Hermans, 2003).

Assuming a multiplicity of inner voices, internal dialogical activity specifically applies to the exchange of thoughts or ideas between at least two I-positions representing specific points of view (Hermans, 1996). Research shows that inner dialogues play an important role in identity construction (e.g., Bhatia, 2002; Hermans and Dimaggio, 2007; Batory, 2010), differentiating and integrating the self as part of the process of self-organization (e.g., Raggatt, 2012; Valsiner and Cabell, 2012), the simulation of social dialogues (e.g., Puchalska-Wasyl et al., 2008; Puchalska-Wasyl, 2011), and general self-reflection and insight (e.g., Markova, 2005; Hermans and Hermans-Konopka, 2010; Rowan, 2011).

Developments within DST (Hermans and Hermans-Konopka, 2010) and associated research (e.g., Oleś and Hermans, 2005; Hermans and Gieser, 2012; Puchalska-Wasyl, 2016; Puchalska-Wasyl et al., 2018) have led to the identification of several forms and functions of internal dialogical activity. For example, Nir (2012) distinguished *contrasting* (or confrontational) and *integrating* dialogues. Contrasting dialogues refer to the clashing of opposing points of view and argumentation until one of them obtains an evident advantage over another. Integrating dialogues tend toward compromising solutions or the integration of opposing points of view into higher levels of abstract meanings. Puchalska-Wasyl (2010) highlighted differences between three forms of dialogical activity: monologue (that implies an interlocutor or audience), dialogue, and changing point of view. This last form refers to the polyphony described by Bakhtin (1973) and Hermans (1996). While dialogue means real exchange of ideas between two or more points of view (I-positions), monologue refers to one-sided communications (whether to oneself or to another person) in which an answer is not expected.

Researchers have recently engaged in efforts to measure individual differences in inner dialogues. For example, the Varieties of Inner Speech Questionnaire (VISQ) (McCarthy-Jones and Fernyhough, 2011; Alderson-Day et al., 2018) measures different phenomenological aspects of inner speech, including a factor on dialogicality (or self-talk occurring as a back-and-forth conversation). Oleś (2009) and Oleś and Puchalska-Wasyl (2012) developed the Internal Dialogical Activity Scale (IDAS), which focuses specifically on the range of different kinds of inner dialogues postulated by DST. Some of the dimensions of this measure include identity, social, supportive, confronting, and ruminative dialogues. The IDAS therefore permits a more thorough examination of DST concepts than the VISQ.

In summary, DST views intrapersonal communication as a complex process of inner dialogues. These dialogues take a wide variety of forms and functions that play important roles in the development of self and identity. However, to date, there has been little research attention devoted to the relationship of these kinds of forms and functions to other kinds of intrapersonal communication. Self-talk appears to be one kind of intrapersonal communication that is similar to inner dialogues.

### Possible Linkages Between Self-Talk and Inner Dialogues

As we noted earlier, the levels of focus are different for the STS and the IDAS. Internal dialogues tend to apply more to a higher level, or meta-features, of intrapersonal communication, compared to the self-regulatory functions assessed by the STS. That is, the STS measures why and when people might talk to themselves, whereas the IDAS primarily assesses the phenomenology of how people talk to themselves.

The potential relationships among self-talk and inner dialogues are theoretically interesting for several reasons. It is conceivable that different kinds of self-talk reflect different I-positions. For example, self-critical self-talk might reveal the presence of confrontational dialogues, whereas self-managing self-talk might be more frequent when people engage in integrative dialogues. Individuals reporting frequent ruminative inner dialogues might also report higher levels of self-critical self-talk.

There are also some likely differences between these two kinds of intrapersonal communication. Self-talk includes a variety of non-dialogical features, such as internal monologues that reflect observations of or commentary on one’s experiences that are not interpersonally or socially directed (e.g., Duncan and Cheyne, 1999; Langland-Hassan and Vicente, 2018) or simple auditory rehearsals (e.g., MacKay, 1992) that do not involve more than one I-position. Thus, it is reasonable to expect that some kinds of self-talk may be unrelated to the frequency of inner dialogues.

Fernyhough (2009, 2016) argues that inner speech is fundamentally dialogic and permits people to take perspectives on, understand, and integrate their internal and external worlds. This process includes creating representations of the inner experiences of other people. As such, it is reasonable to predict that some kinds of self-talk will be positively associated with certain types of inner dialogues. For example, social-assessing self-talk is probably similar to dialogues that include an imagined social mirror.

Some research on the frequency of self-talk is relevant to theoretical conceptions of inner dialogues. For example, Brinthaupt and Dove (2012) found that adults who reported having had an imaginary companion in childhood reported more frequent self-talk than those who did not have one. In addition, they found that adults who grew up as only children without siblings reported more frequent self-talk than those growing up with siblings. Such childhood social experiences might play a role in people’s levels of comfort with, or awareness of, their self-talk as well as the nature of their inner dialogues. Other contributors to the current Research Topic (e.g., Brinthaupt, 2019; Łysiak, 2019) provide additional insights into possible relationships between internal dialogues and self-talk.

### Aims of the Study

Our research examines two specific modes of intrapersonal communication. In particular, we explore the relationships among functions of self-talk and types of inner dialogues in order to clarify the similarities between these modes of intrapersonal communication. Previous research has extensively studied the self-talk and internal dialogue types and functions measured by the STS and IDAS-R. However, no research, to date, has examined the ways that these self-talk and internal dialogue facets relate to and overlap with each other. Brinthaupt et al. (2009) constructed and validated the Self-Talk Scale in the United States, whereas Oleś (2009) published the Internal Dialogical Activity Scale in Poland. In this study, we decided to compare each of these constructs using both United States and Polish samples. We examine the relationships among these two measures through the use of correlational and factor analytic approaches. We are not introducing new ways to assess intrapersonal communication; nor are we primarily interested in cross-cultural differences.

This study explores relationships among the different functions of self-talk defined by the STS and the types of internal dialogues identified by the IDAS. Our general expectation was that individuals who report frequent levels of internal dialogical activity will also report frequent self-talk. However, the strength of these relationships will depend on the specific types and subscales of both kinds of intrapersonal communication. By exploring these relationships, we hoped to better clarify the theoretical and conceptual similarities between self-talk and inner dialogues.

## Materials and Methods

### Participants

Participants were two college student samples. The Polish sample consisted of 181 students (117 women, 64 men), with ages ranging from 18 to 34 (*M* = 24.94, *SD* = 4.24), who attended courses leading to a master’s degree. We drew the United States sample from the university’s General Psychology research pool that was comprised of mostly freshmen and sophomores. This sample consisted of 119 students (66 women, 51 men, two missing), with ages ranging from 18 to 29 (*M* = 19.18, *SD* = 1.86). The two samples differed significantly in age, *t*(297) = 13.92, *p* < 0.001, but did not differ significantly in their gender proportions, *X*^2^(2) = 3.39, *p* = 0.18.

### Measures

#### Self-Talk Scale (STS)

Self-Talk Scale (STS) (Brinthaupt et al., 2009). The STS consists of 16 items, representing the four self-talk functions of self-criticism, self-reinforcement, self-management, and social-assessment. Respondents rate the STS items using a five-point frequency scale (1 = *never*, 5 = *very often*) and using the common stem “I talk to myself when.” Each subscale contains four items. To calculate subscale and total frequency scores, items are summed, with higher scores indicating more frequent self-talk. Research provides good support for the psychometric properties of the STS and the integrity of the four subscales (e.g., Brinthaupt et al., 2009, 2015; Brinthaupt and Kang, 2014).

*Self-criticism* pertains to self-talk about negative events (e.g., “I should have done something differently” and “I feel ashamed of something I’ve done”). *Self-reinforcement* refers to self-talk about positive events (e.g., “I am really happy for myself” and “I want to reinforce myself for doing well”). *Self-management* assesses self-talk about features of general self-regulation (e.g., “I am mentally exploring a possible course of action” and “I want to remind myself of what I need to do”). *Social-assessment* applies to self-talk about people’s future and past social interactions (e.g., “I try to anticipate what someone will say and how I’ll respond to him or her” and “I want to analyze something that someone recently said to me”).

#### Internal Dialogical Activity Scale-R (IDAS-R)

Internal Dialogical Activity Scale-R (IDAS-R). The IDAS-R is a 40-item tool aimed at measuring an overall level of internal dialogical activity as well as eight different kinds of inner dialogues. The original version of the Questionnaire (IDAS) consisted of 47 items and contained seven subscales (Oleś, 2009; Oleś and Puchalska-Wasyl, 2012). Respondents rate the applicability of each item using a five-point scale. In the current revision of the scale, we changed the response format from the original intensity of agreement (1 = *I strongly disagree*, 5 = *I strongly agree*) to a frequency scale (1 = *never*, 2 = *seldom*, 3 = *sometimes*, 4 = *often*, 5 = *very often*). Additional revisions included (1) splitting two complex sentences into simple items containing clear meanings, (2) adding four items, (3) reformulating the wording of several items due to the new response format, and (4) deleting one item as irrelevant.

To test the structure and psychometric properties of the IDAS-R, we collected data from 654 Polish participants (449 women, 205 men) ranging in age from 16 to 80 years (*M* = 31.83, *SD* = 10.93). All participants provided informed consent prior to completing the measure. For the exploratory factor analysis, we used the least squares method for the extraction of factors, with Oblimin rotation and Kaiser normalization. The results provided nine extracted factors, which explained 63% of the variance. However, one of these factors contained low loadings, so we settled on eight factors for the final version explaining 61% of the variance. Each factor consists of five items, resulting in the final 40-item version. We describe the factor scales, their associated internal consistency values, and sample items below.

*Identity Dialogues* refer to questions and answers concerning identity, values, and life priorities (e.g., “Thanks to dialogues with myself, I can answer the question, ‘Who am I?’ and “Through internal discussions I come to certain truths about my life and myself.”). Such dialogues pertain to searching for authenticity and may precede important life choices.

*Maladaptive Dialogues* are internal dialogues treated as undesirable, unpleasant, or annoying (e.g., “I would prefer not to carry on internal conversations” and “The conversations in my mind upset me”). The content and occurrence of such dialogues imply task disturbances or avoidance behavior.

*Social Dialogues* are inner dialogues that reflect future and past conversations (e.g., “When preparing for a conversation with someone, I practice the conversation in my thoughts” and “I continue past conversations with other people in my mind”). These items capture the frequency of continuation of talk with others, preparation for conversation, finishing discussions, or creating alternative conversational scenarios.

*Supportive Dialogues* include intrapersonal communications with persons who have given support and whose closeness is valued (e.g., “When I cannot speak with someone in person, I carry on a conversation with him/her in my mind” and “I carry on discussions in my mind with the important people in my life.”). Such dialogues might provide bolstering of social bonds and help to overcome loneliness by giving support to, and strengthening, the self.

*Spontaneous Dialogues* are inner conversations that occur spontaneously in everyday life (e.g., “I converse with myself and “I talk to myself”). Such dialogues refer to the consideration of different thoughts or opinions as well as a dialogical form of self-consciousness.

*Ruminative Dialogues* consist of dialogues involving self-blame, mulling over failures, and recalling of sad or annoying thoughts or memories (e.g., “After failures, I blame myself in my thoughts” and “I have conversations in my mind which confuse me”). These items capture general rumination tendencies within one’s internal dialogues.

*Confronting Dialogues* are internal dialogues conducted between two sides of the self, such as the “good me” and “bad me” (e.g., “I feel that I am two different people, who argue with each other, each wanting something different” and “I argue with that part of myself that I do not like”). Such internal disputes imply a sense of incoherence, polarization, or even fragmentation of the self.

*Change of Perspective* refers to changes in point of view in service of understanding challenging situations or searching for solutions (e.g., “When I have a difficult choice, I talk the decision over with myself from different points of view” and “In my thoughts I take the perspective of someone else”). Such dialogues might involve taking a fruitful or conflicted perspective of another person.

For each of these subscales, summing the five items creates a total score, with higher scores indicating greater frequency of that kind of dialogue. It is also possible to compute an overall inner dialogue score by summing the ratings of all 40 items. In the current study, this total score, called Internal Dialogical Activity reflects a person’s general frequency of engagement in internal dialogues.

### Procedure

We created two parallel Polish and English language versions of the measures. For the STS, one of the research team members who speaks both Polish and English first translated the scale into Polish. A different colleague then back translated the Polish STS version to English. A native English speaking team member reviewed this version and indicated any areas of clarification, confusion, and discrepancy. We then created the final Polish version of the STS. For the IDAS-R, a team member translated the original (Polish) version of the measure into English. A native English-speaking team member then reviewed this version for clarity. A team member then back translated this version into Polish and identified any discrepancies or areas of confusion. We then implemented necessary corrections to create the final English version of IDAS-R.

The study received approval from the Institutional Review Board (IRB), Middle Tennessee State University, United States. Participants provided their written informed consent when the institution required it. They completed the main measures in counterbalanced order individually or in small groups of 5–10 people. Demographic items appeared at the end of the survey.

## Results

Descriptive statistics for both samples appear in Table 1. As the table shows, the alpha coefficients for the STS and IDAS-R were similar across the United States and Polish samples, with comparable and acceptable values. Both samples also showed similar patterns in the relative frequency of the four types of self-talk, with self-managing self-talk most common and self-reinforcing self-talk least common. Among the IDAS-R facets, both samples reported relatively low levels of maladaptive and confronting dialogues and relatively high levels of social and spontaneous dialogues.

**TABLE 1:** Descriptive statistics for the self-talk scale and the internal dialogical activity scale—revised for United States, polish, and combined samples.

Scale	United States sample (*n* = 119)				Polish sample (*n* = 181)				Combined sample (*n* = 300)			
	α	*M*	*SD*	95% CI	α	*M*	*SD*	95% CI	α	*M*	*SD*	95% CI
STS total score	0.88	59.97	10.98	[57.98, 61.97]	0.88	49.92	12.64	[48.06, 51.78]	0.90	53.92	12.96	[52.44, 55.40]
Social-assessment	0.70	14.86	3.42	[14.24, 15.48]	0.83	12.04	4.61	[11.36, 12.72]	0.82	13.16	4.39	[12.66, 13.66]
Self-Reinforcement	0.85	13.56	3.88	[12.86, 14.27]	0.86	11.58	4.29	[10.95, 12.21]	0.86	12.37	4.24	[11.89, 12.85]
Self-Criticism	0.83	15.13	3.79	[14.44, 15.81]	0.73	12.22	3.78	[11.67, 12.78]	0.79	13.38	4.04	[12.92, 13.84]
Self-Management	0.73	16.43	3.03	[15.88, 16.98]	0.79	14.07	4.07	[13.47, 14.67]	0.79	15.01	3.86	[14.57, 14.45]
IDAS-R total score	0.94	119.78	26.90	[114.90, 124.66]	0.95	108.59	29.45	[104.26, 112.93]	0.95	113.05	28.94	[109.75, 116.34]
Identity dialogues	0.78	15.87	4.48	[15.05, 16.68]	0.87	14.75	5.20	[13.99, 15.51]	0.841	15.19	4.94	[14.63, 15.76]
Maladaptive dialogues	0.62	11.32	3.79	[10.63, 12.01]	0.70	10.75	4.04	[10.15, 11.34]	0.681	0.97	3.94	[10.52, 11.42]
Social dialogues	0.72	17.58	4.15	[16.83, 18.33]	0.84	17.81	5.00	[17.08, 18.55]	0.80	17.72	4.68	[17.19, 18.25]
Supportive dialogues	0.81	13.82	4.72	[12.96, 14.67]	0.86	13.28	5.45	[12.48, 14.08]	0.841	3.49	5.17	[12.90, 14.08]
Spontaneous dialogues	0.79	17.13	4.34	[16.35, 17.92]	0.861	4.90	5.29	[14.12, 15.67]	0.84	15.78	5.05	[15.21, 16.36]
Ruminative dialogues	0.771	5.22	4.44	[14.41, 16.02]	0.81	13.33	4.88	[12.62, 14.05]	0.80	14.08	4.79	[13.54, 14.62]
Confronting dialogues	0.76	13.13	4.73	[12.27, 13.98]	0.831	1.35	5.03	[10.62, 12.09]	0.81	12.06	4.98	[11.49, 12.62]
Change of perspective	0.70	15.72	4.01	[14.99, 16.45]	0.79	12.36	4.50	[11.70, 13.02]	0.79	13.69	4.61	[13.17, 14.22]

Comparison of the two samples revealed that the United States students reported significantly higher scores than their Polish peers on the total STS [*t*(297) = 7.09, *p* < 0.001, *g* = 0.84] as well as the social-assessment [*t*(297) = 5.71, *p* < 0.001, *g* = 0.67], self-reinforcement [*t*(297) = 4.06, *p* < 0.001, *g* = 0.48], self-criticism [*t*(297) = 6.49, *p* < 0.001, *g* = 0.77], and self-management [*t*(297) = 5.40, *p* < 0.001, *g* = 0.64] STS subscales. A similar pattern emerged for overall IDAS-R and five of its eight subscales. In particular, United States students reported higher scores than the Polish students on the total IDAS-R [*t*(297) = 3.33, *p* < 0.001, *g* = 0.39], as well as the identity [*t*(297) = 1.92, *p* < 0.05, *g* = 0.23], spontaneous [*t*(298) = 3.84, *p* < 0.001, *g* = 0.45], ruminative [*t*(298) = 3.40, *p* < 0.001, *g* = 0.40], confronting [*t*(298) = 3.06, *p* < 0.002, *g* = 0.36], and change of perspective [*t*(298) = 6.61, *p* < 0.001, *g* = 0.78] dialogues.

Table 2 reports the correlations among the STS and IDAS-R measures for each sample and indicates those correlations that reached the 0.001 level of significance. The correspondence between these two kinds of intrapersonal communication turned out to be consistently positive, with most correlations in the moderate to strong range. For the Polish sample, 36 of the 44 correlations between the STS and IDAS-R total and subscale scores were significant. For the United States sample, 35 of 44 of these correlations were significant. In the Polish sample, significant correlations ranged between 0.24 and 0.59; in the United States sample, significant relationships ranged between 0.29 and 0.62. Moreover, the patterns of relationships in both samples were similar. Total STS and IDAS-R scores correlated 0.56 in the Polish sample and 0.62 in the United States sample.

**TABLE 2:** Correlations between the STS and IDAS-R: results from Polish sample above the diagonal and for United States sample below the diagonal.

PL														
US	1	2	3	4	5	6	7	8	9	10	11	12	13	14
1 Social-assessment		0.31*	0.37*	0.71*	0.33*	0.02	0.42*	0.46*	0.45*	0.42*	0.27*	0.39*	0.81*	0.47*
2 Self-reinforcement	0.27		0.26*	0.44*	0.37*	0.07	0.11	0.26*	0.40*	0.20	0.21	0.32*	0.67*	0.33*
3 Self-critical	0.55*	0.45*		0.44*	0.24*	0.21	0.24*	0.25*	0.35*	0.45*	0.29*	0.33*	0.67*	0.40*
4 Self-management	0.71*	0.42*	0.48*		0.41*	–0.01	0.38*	0.38*	0.56*	0.41*	0.31*	0.45*	0.86*	0.50*
5 Identity dialogues	0.43*	0.32*	0.31*	0.57*		–0.03	0.41*	0.50*	0.74*	0.58*	0.44*	0.72*	0.45*	0.75*
6 maladaptive dialogues	0.14	0.12	0.32*	0.19	0.34*		–0.01	0.18*	0.09	0.34*	0.56*	0.28*	0.09	0.38*
7 Social dialogues	0.62*	0.09	0.27	0.56*	0.52*	0.17		0.62*	0.50*	0.51*	0.30*	0.42*	0.39*	0.65*
8 Supportive dialogues	0.48*	0.29*	0.40*	0.49*	0.65*	0.33*	0.64*		0.61*	0.68*	0.51*	0.56*	0.45*	0.80*
9 Spontaneous dialogues	0.45*	0.34*	0.42*	0.56*	0.65*	0.27	0.57*	0.64*		0.62*	0.55*	0.76*	0.59*	0.84*
10 Ruminative dialogues	0.50*	0.14	0.53*	0.42*	0.55*	0.51*	0.50*	0.64*	0.57*		0.64*	0.67*	0.49*	0.85*
11 Confronting dialogues	0.27	0.28	0.40*	0.31*	0.51*	0.57*	0.32*	0.60*	0.53*	0.62*		0.71*	0.36*	0.78*
12 Change of perspective	0.54*	0.36*	0.42*	0.60*	0.68*	0.41*	0.64*	0.66*	0.70*	0.70*	0.62*		0.49*	0.87*
13 STS Total	0.79*	0.71*	0.81*	0.81*	0.51*	0.30	0.47*	0.53*	0.56*	0.50*	0.41*	0.60*		0.56*
14 IDAS-R Tot	0.56*	0.32*	0.50*	0.60*	0.80*	0.57*	0.71*	0.84*	0.80*	0.82*	0.78*	0.87*	0.62*	

United States sample: *n* = 119; Polish sample: *n* = 181; *p < 0.001.

On the one hand, these results show moderate, positive relationships between several self-talk functions and types of internal dialogues. On the other hand, there is evidence of possible independence of these kinds of intrapersonal communication. For our next set of analyses, we sought to determine the extent of independence of STS and IDAS-R subscales. We used both canonical correlational and exploratory factor analysis with the combined samples to address this question.

To answer the question of overlap between the two measures of intrapersonal communication, we first used canonical correlational analysis, which permitted us to explore mutual relationships between STS and IDAS-R subscales in a more complex and advanced way. This analysis allows us to find features that are important for explaining the covariation between the subscales of the STS and IDAS-R. We conducted the analysis on the combined samples with each participant represented by their scores on the four STS and the eight IDAS-R subscales. Because of the potential negative effects of outliers on CCA, we first eliminated respondents who scored three standard deviations above or below the mean on the total score of either measure. This resulted in a new sample size of 293 (180 women, ages 18–34). The results of this analysis showed three significant canonical correlations: 0.64, 0.43, and 0.33 (all *p* < 0.001), explaining, respectively, 41%, 19%, and 11% of the variance (see Table 3). The first canonical variable represented over half of the variance from the original set of variables and explained about 25% of the variance from the opposite set of variables.

**TABLE 3:** Canonical correlations between the IDAS-R and STS.

Methods and scales			Canonical variables		
			1	2	3
IDAS-R	Identity dialogues		–0.68	–0.40	–0.04
	Maladaptive dialogues		–0.21	0.30	–0.53
	Social dialogues		–0.57	0.13	0.67
	Supportive dialogues		–0.67	–0.06	0.27
	Spontaneous dialogues		–0.90	–0.28	–0.10
	Ruminative dialogues		–0.82	0.43	–0.11
	Confronting dialogues		–0.60	0.05	–0.32
	Change of perspective		–0.91	–0.14	–0.10
		*% Var IDAS-R*	49	7	12
		*% Var STS*	20	1	1
		*CR*	0.64	0.44	0.33
		*p*<	0.001	0.001	0.001
		*CR*^2^	0.41	0.19	0.11
STS	Social assessment		–0.84	0.09	0.45
	Self-criticism		–0.75	–0.43	–0.50
	Self-reinforcement		–0.54	0.62	–0.40
	Self-Management		–0.90	–0.24	0.14
		*% Var STS*	59	16	16
		*% Var IDAS*	25	3	2

Interestingly, all loadings were negative, with lack of self-talk functions (see canonical loadings) corresponding to reduced inner dialogues of all kinds. However, according to the reversed loadings, this variable represented the presence of four self-talk functions, namely, self-management, social assessment, self-criticism, and, to a lesser degree, self-reinforcement, and almost all types of inner dialogues. This variable can be labeled “dialogical self-talk.” The second and third canonical variables represented only a small amount of residual variance from the original variables (both 16%) and explained very little of the residual variance (3% and 2%) from the opposite set of variables.

In order to examine similarities of both kinds of intrapersonal communication, we also used exploratory factor analysis, principal components with Varimax rotation, and the Scree test for factor extraction. The 12 subscales (four STS, eight IDAS-R) served as the variables in this analysis. We identified a four-factor solution, according to the Scree test. The four extracted factors explained 79% of the variance (for loadings see Table 4).

**TABLE 4:** Results of EFA: loadings for four-factor solution.

Scale/Variable	Factor I	Factor II	Factor III	Factor IV
Social dialogues	0.79	0.32	–0.06	–0.23
Supportive dialogues	0.78	0.23	0.22	0.04
Identity dialogues	0.74	0.05	0.08	0.49
Spontaneous dialogues	0.71	0.26	0.15	0.44
Change of perspective	0.66	0.24	0.38	0.41
Ruminative dialogues	0.64	0.33	0.50	0.06
Social assessment	0.40	0.82	–0.00	0.09
Self-management	0.35	0.74	–0.02	0.34
Self-criticism	0.07	0.73	0.37	0.21
Maladaptive dialogues	0.02	0.05	0.91	–0.03
Confronting dialogues	0.47	0.09	0.71	0.24
Self-reinforcement	0.05	0.34	0.06	0.81

The factors explained 49.3%, 11.7%, 8.9%, and 7.2% of the variance, respectively. Factor 1 (Internal Dialogicality) represented the different kinds of IDAS-R inner dialogues except for maladaptive and confronting dialogues. This factor explained almost half of the variance in the data, with six of the 12 subscales having relatively high loadings on it. Regarding the content of this factor, the IDAS-R subscales related to contact and union with the self’s and others’ inner dialogues, representing the adaptive side of inner dialogues. Interestingly, the STS functions did not load strongly on this factor.

Factor 2 (Self-Regulatory Self-Talk) contained three STS subscales/functions: Social Assessment, Self-Management, and Self-Criticism. These subscales seem to represent self-talk aspects that are different from the types of internal dialogues. Factor 3 (Disruptive Dialogicality) contained the maladaptive and confronting IDAS-R subscales. These types of inner dialogues represent a kind of psychic burden caused or accompanied by unpleasant or tension producing dialogues. Factor 4 (Self-Enhancing Self-Talk) included only the Self-Reinforcement STS subscale.

Summing up, both CCA and EFA showed some overlap between self-talk and inner dialogical activity. However, the results are not strong enough to identify these two modes of intrapersonal communication as variable aspects of the same phenomena. Instead, they seem to be complementary types of intrapersonal communication that serve different functions.

## Discussion

This purpose of this study was to examine the similarities between two kinds of intrapersonal communication using two recent multidimensional measures of inner dialogue and self-talk. As we expected, there were moderate to strong relationships among the total and subscale scores of the IDAS-R and STS. These results suggest that internal dialogical activity shares a good deal of variance with common self-talk functions. In other words, there is a significant self-talk component to internal dialogues. Although Brinthaupt et al. (2009) developed the STS independently of Dialogical Self Theory, the self-regulatory functions identified by their measure provide some conceptual and theoretical support for that theory.

Both the zero-order correlational data as well as the canonical correlations showed significant relationships between the self-talk functions and the types of inner dialogues. The results generally showed STS and IDAS-R overlap of between 30% and 40%. The common variance of the subscales of STS and IDAS-R, according to the canonical correlation analysis, was about 41%. Such results show that self-talk functions and inner dialogue types are, on the one hand, clearly related variables.

On the other hand, there are elements of each kind of intrapersonal communication mode that are different. For example, the STS functions appear to represent dynamic aspects of intrapersonal communication, involving active processing of current or recent situations and compensation for behavioral challenges and cognitive disruptions (see Brinthaupt, 2019, this Research Topic). Alternatively, different types of inner dialogical activity seem to represent contemplative aspects of intrapersonal communication, such as reflecting about oneself or deliberating about different facets of one’s identity. The types of inner dialogues illustrate qualities of awareness of human consciousness: representations of others in one’s mind, overcoming of loneliness, keeping bonds with significant others, fighting for autonomy, and controlling of a social mirror (e.g., Puchalska-Wasyl et al., 2008; Rowan, 2011; Stemplewska-Żakowicz et al., 2012; Valsiner and Cabell, 2012).

Research on self-esteem suggests that inner dialogues and self-talk serve possibly different roles. Oleś et al. (2010) found that total and subscale IDAS scores correlated negatively and significantly with self-esteem. However, Brinthaupt et al. (2009) found that self-esteem did not correlate significantly with total and subscale STS scores (except for self-critical self-talk). Both studies measured self-esteem with the same tool, Rosenberg’s Self-Esteem Scale, but collected data from different populations/countries (Poland and the United States).

In the present study, there was evidence for more frequent intrapersonal communication activity in the United States sample, especially with respect to the self-talk functions. It is not clear whether these results reflect cultural or age differences between the two samples. The American students were a few years younger than the Polish participants. It is conceivable that younger people might engage in more intrapersonal communication (both IDAS-R and STS) than older people. If younger adults experience the uncertainty of adult life (Hermans and Hermans-Konopka, 2010) and engage more frequently in identity construction processes during late adolescent and emerging adulthood (Arnett, 2000; Hermans and Dimaggio, 2007), then we would expect increases in reports of inner dialogues and self-talk.

Cultural differences between the United States and Polish samples might also account for the differences in reported frequency of self-talk and inner dialogues. Research shows that higher identity integration is associated with less frequent internal dialogical activity measured by the IDAS (Oleś, 2011) and that higher self-concept clarity integration is associated with less frequent internal dialogical activity (Oleś et al., 2010). If the two samples differed in their identity or self-concept clarity (something that could be associated with the age differences), this might account for the frequency differences we observed on the STS and IDAS-R. Thus, exploring age and cultural differences in intrapersonal communication appears to be a fruitful avenue for future research.

### Limitations and Implications for Future Research

We operationalized aspects of intrapersonal communication using two self-report measures. As such, this study’s data refer mainly to aspects of internal dialogue and self-talk that respondents are consciously aware of or can access upon reflection. As others (e.g., Beck, 1976) have noted, not all intrapersonal communication is conscious, and the present measures are limited to those situations and experiences that respondents are able to recall or infer based on other information. In addition, the list of functions and types of self-talk and internal dialogues tapped by the STS and IDAS-R is not exhaustive. For example, the STS does not measure the frequency of self-distancing and adaptive coping that have been shown to be implicit functions of third-person self-talk (Kross et al., 2014) or the generic “you” that is used for general meaning making to help “people ‘normalize’ negative experiences by extending them beyond the self” (Orvell et al., 2017, p. 1299). There may be additional cognitive, motivational, or emotional functions not tapped by the STS and IDAS-R (e.g., Alderson-Day et al., 2018; Latinjak et al., 2019).

We believe that methodological artifacts are unlikely to explain the results. The factor analysis loadings do not reflect solely positive and negative valenced items from the measures. For example, ruminative dialogues appeared within Factor 1, and self-critical self-talk appeared in Factor 2. The results appear to map more closely to the overall frequency of use of each kind of intrapersonal communication, with the three least frequent facets (maladaptive and confronting dialogues and self-reinforcing self-talk) emerging as separate, minor factors. In addition, both scales used the same response format, which should reduce response artifacts. However, the STS uses a specific instructional prompt (“I talk to myself when…” certain situations occur), With the IDAS-R, participants rate statements related to self- and other-related dialogical thinking situations. Thus, there is a distinction between *when* one talks to oneself (STS) and *how* one talks to oneself (IDAS-R). Future research is needed for a careful and systematic examination of the item content and construct indicators of the STS and IDAS-R.

Because the STS and IDAS-R have semantically overlapping item content, it is important to examine the predictive value of each scale with external criteria. Although we have yet to examine external criteria that might address the differentiation of self-talk and inner dialogues, there is some evidence that internal dialogues are more strongly related to self-esteem than is self-talk (Brinthaupt et al., 2009; Oleś et al., 2010), suggesting potential differences in the functions served by these two kinds on intrapersonal communication. Studying the operation of internal dialogues and self-talk in specific self-regulatory contexts (e.g., novel or stressful situations) could provide additional insight into the predictive value and overlap of the measures.

Future research will need to continue examining the structure and properties of the STS and IDAS-R. One possible direction is to examine situation-specific intrapersonal behavior. For example, within specific contexts or situations (e.g., coping with stress, arriving at a decision, or construing personal identity), there may be specific behavioral signatures (Mischel and Shoda, 1995) containing different combinations of internal dialogue or self-talk types. As the contributions to this Research Topic show, there are other kinds of intrapersonal communication beyond internal dialogues and self-talk. Exploring the relationships among the varieties of intrapersonal communication would also be a worthy goal for future research.

## Conclusion

We have shown that the relationship between inner dialogue and self-talk is interesting and complex and that the study of this relationship is a theoretically valuable research goal. There are several additional modes, categories, and functions served by, or relevant to, intrapersonal communication (e.g., Heavey and Hurlburt, 2008). Researchers might find it profitable to utilize the IDAS-R and the STS to explore further the overlap between, and distinctions among, these phenomena.

## Data Availability Statement

The datasets generated for this study are available on request to the corresponding author.

## Ethics Statement

The studies involving human participants were reviewed and approved by the Institutional Review Board (IRB), Middle Tennessee State University, United States. This study spanned over two countries. The patients/participants provided their written informed consent to participate in this study when it was required by the national legislation and the institutional requirements.

## Author Contributions

PO conceptualized the research, led the empirical research in Poland, and revised and wrote the first version of manuscript. TB prepared the idea of research, led the empirical research in United States, finally edited, revised, and corrected the first version of manuscript. RD conducted the empirical research in the United States and prepared the database. DP, conducted the empirical research in Poland, prepared the database, and performed computations.

## Conflict of Interest

The authors declare that the research was conducted in the absence of any commercial or financial relationships that could be construed as a potential conflict of interest.
